# Molecular diagnostics in medical mycology

**DOI:** 10.1038/s41467-018-07556-5

**Published:** 2018-12-03

**Authors:** Brian L. Wickes, Nathan P. Wiederhold

**Affiliations:** 1 The Department of Microbiology, Immunology, and Molecular Genetics, The University of Texas Health Science Center at San Antonio, 78229 San Antonio, TX USA; 2 The Fungus Testing Laboratory, Department of Pathology and Laboratory Medicine, The University of Texas Health Science Center at San Antonio, 78229 San Antonio, TX USA

## Abstract

Diagnosing fungal infections poses a number of unique problems, including a decline in expertise needed for identifying fungi, and a reduced number of instruments and assays specific for fungal identification compared to that of bacteria and viruses.These problems are exacerbated by the fact that patients with fungal infections are often immunosuppressed, which predisposes to infections from both commonly and rarely seen fungi. In this review, we discuss current and future molecular technologies used for fungal identification, and some of the problems associated with development and implementation of these technologies in today’s clinical microbiology laboratories.

## Abstract (web summary)

Diagnosing fungal infections poses a number of unique problems. In this Review, Wickes and Wiederhold discuss molecular technologies used for fungal identification, and the problems associated with their development and implementation in today’s clinical microbiology laboratories.

## Introduction

Molecular diagnostic assays generally consist of methods and/or instruments that detect biomarkers found in the DNA, RNA, or gene products of an organism^1,2^. Historically, molecular diagnostic assays for invasive infections have been labor intensive and required a high degree of technical expertise. Although late to the suite of current clinical molecular diagnostics, fungal assays are being developed and commercialized at an increasing rate; however, fungi offer unique challenges to assay development, which are compounded by the general challenges facing new diagnostic tests. These challenges center around the need to quickly and accurately identify the infecting species from a growing list of fungal pathogens recovered from a variety of patient types and clinical specimens. In fact, time to diagnosis is one of the most important risk factors for mortality from many of the systemic mycoses^3,4^. Thus, rapid diagnosis is a critical component of patient care. Unfortunately, phenotypic and biochemical identification methods are often time consuming, which has created an increasing demand for new molecular diagnostic methods for fungal identification.

In this review, we discuss some of the challenges to the development of molecular diagnostics, including those that are unique to fungi. Among these challenges are sample preparation, which is typically more difficult than for bacterial and viral agents, and reference samples (cultures, sequences, etc.), which can be difficult to access. Developing new diagnostic strategies can be challenging enough at the bench, however, the path to implementation of these strategies in a clinical microbiology laboratory has many hurdles to overcome prior to approval for use in clinical settings, which need to be considered early in the development process. Finally, we highlight some of the technologies and platforms that form the basis for new assay development including some of the newer technologies based on genome sequencing.

## Continued diagnostic urgency due to new and old fungal pathogens

### Systemic mycoses continue to be a public health threat

It is estimated that more than 1 billion people throughout the world have fungal infections^5,6^ with 15–30% of these infections being serious^5,7^. While the number of fungal species pathogenic for humans is estimated to be approximately 300^8^, at our institution we have identified >1500 different fungal species isolated from human specimens. The increase in the number of pathogenic fungi has tracked with the increasing susceptibility of patients, due primarily to the length and severity of immunosuppression, in addition to aggressive medical treatment that patients may experience today. Patient groups at high risk include those with AIDS, those receiving immunosuppressive chemotherapy, transplant recipients, certain surgeries, and those in intensive care settings^9,10^. Unfortunately, as new life-saving medical procedures are developed and employed, additional fungi not seen before are recovered from patients with increasing frequency, and well known fungal pathogens continue to cause a significant number of infections.

Yeasts (see Box 1: Glossary) that are commonly associated with invasive mycoses include *Cryptococcus* and *Candida* species. New cases of cryptococcosis are estimated to exceed more than 500,000 per year^11^ with most new cases being found in sub-Saharan Africa. Unfortunately, in this region, cryptococcosis may kill more AIDS patients annually than tuberculosis^12^, which reflects deficiencies in both diagnosis and treatment in undeveloped countries. *Candida* species are the most common fungal pathogen in humans and are associated with significant morbidity, mortality, and increased healthcare costs^13^. In fact, mortality due to candidemia may increase by 20% after only a 12-hour delay in the initiation of treatment following collection of the initial positive blood culture^14^. Additionally, drug resistance has become so common that members of this genus have been placed on the World Health Organization and Centers for Disease Control Antibiotic threat lists^15,16^. Consequently, new molecular diagnostic assays are also needed to rapidly detect drug resistance.

For both *Candida* and *Cryptococcus* species the epidemiologic profile is changing, with new members of both genera being recovered more frequently from patients^17,18^. These changes place a burden on microbiology laboratories to keep up with current infection trends. For example, a new species of *Candida* (*C. auris*) has only recently been discovered^19^ and is notable because of its unusually high mortality rate due to a combination of factors, including frequent multidrug resistance and misidentification by commonly used diagnostic assays^20^. These observations combined with the rapid emergence and global spread has resulted in the issuance of a public health alert in the US^21^.

Many molds are also major systemic pathogens that frequently cause life-threatening infections. In solid organ transplant recipients and stem cell transplant recipients, for example, mortality rates associated with *Aspergillus* infections can range between 40% to 75%^22^, and approach 90% in certain high risk patients^23^. One of the most crucial predictors of clinical outcome in patients with acute invasive aspergillosis is time to diagnosis, which is particularly challenging for the *Aspergilli* because these fungi rarely circulate in the bloodstream, making detection by traditional blood culture difficult. Other diagnostic methods such as imaging or histopathology are insensitive and nonspecific, or invasive in the case of biopsy. Even PCR, which is not dependent on live cells, can be insensitive depending on the template source^24^. In addition, clinical sensitivity may be reduced in patients receiving antifungal agents due to the reduction in the targets used by molecular assays to detect pathogens. This issue may further complicate diagnosis of invasive fungal infections in patients receiving antifungal prophylaxis or empiric therapy. Furthermore, because the *Aspergilli* are among the most ubiquitous fungi in the environment, great care must be taken to avoid false positives due to contamination. In fact, fungal contamination of PCR reagents, including polymerases, is well known^25^.

Finally, while a substantial number of systemic mycoses occur in patients with some type of immunosuppression, numerous fungi can cause disease in healthy individuals. These infections can be difficult to diagnose if a patient is not from an endemic area where they were infected, such as in cases of coccidioidomycosis, due to lack of familiarity with the fungus or lack of suspicion of a specific fungus as an etiologic agent. Other fungi can have nonspecific symptoms and often require a patient history to direct the selection of a specific diagnostic assay. For example, in a story that received international coverage, members of a Thailand boys soccer team that were trapped in a cave were hospitalized immediately after rescue, due in part to concerns that they were at risk for histoplasmosis^26^. *Histoplasma capsulatum* infections can be very slow and require 1-2 weeks or more of incubation. In the case of these patients, histoplasmosis is a well-known disease that can be contracted by people who spend time in caves. If they had become symptomatic, they could have been quickly tested for *H. capsulatum* using a specific assay. In the absence of their recent history, diagnosis could take much longer and would likely require a differential diagnostic process prior to selection of a confirmatory assay.

### Box 1. Glossary

Anamorph: The asexual phase of a fungal lifecycle.

Conformitè Europèenne In Vitro Diagnostic (CE-IVD): European Union designation that is equivalent to US FDA approval. CE-marked devices used for medical diagnostics indicate that a device or assay has been certified by the manufacturer to meet the essential legal requirements for safety and performance when used as indicated by the manufacturer. CE-marked products can be marketed anywhere in the European Union, however, a CE-marked diagnostic does not have automatic reciprocity for use in the US.

Dermatophyte: Class of fungi consisting of species from multiple genera (such as *Microsporum*, *Trichophyton*, and *Epidermophyton*) that cause diseases of the skin, hair, and nails. Infections are usually superficial and can be spread by direct contact from infected humans, contaminated soil, or animals.

Dimorphism: Ability of a fungus to exist in different morphologies (such as yeast and hyphae) during normal vegetative growth.

Food and Drug Administration (FDA): a federal agency within the U.S. Department of Health and Human Services. Among the major responsibilities of the agency are the regulation of food, drugs, vaccines, medical devices, cosmetics, dietary supplements, radiation-emitting devices, and tobacco.

Fungemia: Presence of fungi in the blood.

GenBank: The U.S. National Institutes of Health open access, annotated, nucleic acid sequence database and corresponding protein translations, if applicable. The database is maintained by the National Center for Biotechnology Information and receives deposits from contributors around the world.

Hyphae: Long, branched, multicellular, filamentous, or tubular structures produced by fungi. Hyphae grow from the tip outward and are responsible for absorbing food from the substrate when the fungus is growing vegetatively. Hyphae can also be produced during the sexual phase of the fungus, when they differentiate to produce sexual structures that can be important for distinguishing a fungus taxonomically.

Mold: A fungus that is growing as hyphae, usually with a filamentous appearance.

Mucorales: Order in the phylum Zygomycota. They are generally fast-growing molds that have hyphae lacking septa. Asexual spores (sporangiospores) are produced in sac-like structures called sporangia, which are produced on a specialized hyphal structure called a sporangiophore.

Mucormycosis: A fungal infection caused by members of the Mucorales. These infections are sometimes also called zygomycosis, a broader term since zygomycosis would include members of all orders within the phylum Zygomycota. The most common human pathogens in this order are members of the genus *Rhizopus*, *Mucor*, *Lichtheimia* (formerly *Absidia*), and *Cunninghamella*.

Premarket approval (PMA): A type of FDA regulatory review that is the most stringent type of marketing application required prior to marketing a medical device. A PMA often involves a new device (typically Class III) that has no similarity to a previously legally marketed (predicate) device. The purpose of the PMA is to demonstrate to the FDA that the device is safe and effective.

Real-time PCR or quantitative real-time PCR (qPCR): Polymerase chain reaction methods that monitor the amplification of template nucleic acid as it happens in real-time, i.e., with each amplification cycle, instead of at the end of the reaction. Depending on the chemistry and strategy employed in the PCR reaction, real-time PCR can be quantitative or semi quantitative.

Teleomorph: Sexual reproductive stage of a fungal lifecycle.

Type culture: A viable culture of a fungus that is directly descended from the material that the original description of the species, or other taxonomic classification, was based upon.

Yeast: A unicellular fungus that is round, oval, or similarly shaped. Some species of fungi can produce yeast cells that can become elongated, resembling hyphae (pseudohyphae). Asexual reproduction of yeast cells occurs mitotically by budding.

Whole-genome sequencing (WGS): This method is sometimes referred to as next generation sequencing, after earlier generations defined by Sanger sequencing and capillary sequencing technologies. There are variations in WGS chemistry depending on platform but most use massively parallel sequencing by synthesis, which detects and discriminates single bases as they are incorporated into growing nucleotide chains. Because of the speed of sequencing and reduction in cost from capillary sequencing, data from an entire genome can be quickly produced after a single run, which facilitates much deeper analysis.

510(k) clearance: Requirement by the FDA of manufacturers to notify the FDA in advance of an intent to market a medical device. It serves as premarket notification that is ultimately used for marketing clearance of the device by the FDA. Approval is given if FDA determines that the device is as safe or effective as current legally marketed devices or standards recognized by the FDA that are used for the same purpose.

### Replacing classical mycological expertise

In addition to the major human fungal pathogens, less frequently encountered fungi pose special problems because routine clinical laboratories may lack the expertise, sophisticated equipment, or technologies that enable the correct identification. For example, an outbreak of fungal meningitis in patients receiving steroid preparations contaminated with the rare pathogen *Exserohilum rostratum* resulted in 750 infected patients and 61 deaths^27^ (Box 2). The etiologic agent was identified only after a clinician made the link between a case of fungal meningitis and an epidural injection a number of weeks earlier^28^. Similarly, in 2011 a tornado hit Joplin, Missouri, sending contaminated debris into the air that struck numerous people. Many of the wounds became infected with a soil fungus called *Apophysomyces trapeziformis*^29^, leading to numerous cases of necrotizing mucormycosis. Identification ultimately required clinicians to recognize similar symptoms among multiple patients, leading them to request and coordinate a response from the Centers for Disease Control and Prevention that eventually resulted in the identification of the fungus. Unfortunately, 13 people were infected and 5 died from the infection^29^. These cases illustrate a major problem in medical mycology, which is the absence of expertise and tools needed to rapidly identify an infecting fungus, particularly those that are less commonly encountered.

One solution to this problem is improving our diagnostic platforms in order to identify infecting fungi quicker and with better accuracy and precision. With the continued growth of the molecular diagnostic field, new platforms are being developed and approved for diagnostic use. There is, however, an important caveat to this approach. As technologies become better at filling the gap of fungal diagnosis, already diminished training programs in mycology, particularly in fungal identification, are becoming further depleted. This problem was recognized many years ago but has gone largely unaddressed^3,30^. Given the vast diversity of fungal species that are capable of causing disease in humans and the relatively few numbers of organisms that have been targeted with newer technologies, there is a continued need for clinical mycologists skilled at identification based on morphologic and phenotypic characteristics.

### Box 2. Rare and emerging fungi

A contemporary problem of fungal diagnostics is that, like all fields of medical microbiology, medical mycology is somewhat dynamic in that new organisms constantly need to be accounted for by both clinicians and the microbiology laboratory. On the one hand, rare fungal pathogens, which might have been known for many years to be pathogens, are problematic because it is generally not cost effective to design assays that can identify most or all of the human fungal pathogens since the vast majority will never be seen in the laboratory or in patients. On the other hand, new pathogenic fungi regularly are identified and if they are serious enough, such as in the recently emergent *Candida auris*, assay developers need to determine if a new species needs to be added to the list that a specific assay can detect. If so, they need to obtain these species and adapt their assay for their identification, and then demonstrate this capability by validating the assay on these organisms. *Exserohilum rostratum* is an example of a rare fungal pathogen with less than 50 reported cases prior to the steroid injection outbreak^27^, even though the fungus has been known for 50 years. Rare fungal infections may not commonly occur because a suitable host for the fungus may only be rarely encountered, however, once a host population with a favorable susceptibility intersects with an opportunistic fungus, infection can occur. In the case of *Exserohilum rostratum*, this fungus only became widespread due a common infection route; injection of contaminated methylprednisolone acetate, usually via the epidural route. This outbreak ultimately became the largest healthcare-associated outbreak in US history^99^. *Apophysomyces trapeziformis* is an example of an emerging human fungal pathogen. It was only recently described in 2010^100^, with infections being characterized by aggressive tissue necrosis. The fungus is a member of the Mucorales and is now recognized as a pathogen of trauma patients due, in part, to the Joplin tornado. Other members of this genus also cause infections in trauma patients in addition to other patients with specific types of immunosuppression. Although identification of these two fungi is uncomplicated, they are difficult to identify in routine clinical laboratories because laboratory personnel might never see them in their lifetime. Outside of a panfungal sequence-based assay, neither organism would likely be on a list that a new fungal identification assay would target.



## Challenges for new fungal diagnostic assays

### Desirable assay characteristics

There are several criteria that are preferred when developing molecular diagnostic assays (Table 1)^31–35^. For assays that rely on specialized or advanced instruments, equipment cost, maintenance, size, and installation requirements may determine if the particular platform is purchased. An expensive instrument should be expandable to handle large numbers of specimens and versatile enough to detect and identify unrelated organisms or be capable of other applications in order to be cost effective. Those that rely on databases for identification must be able to incorporate new organisms into the diagnostic capability and should be regularly updated. Outputs of a positive or negative result, name of the organism, or a numerical result with clearly established cutoff values for detection and/or identification are crucial. Post-assay data manipulation is undesirable as it offers the opportunity to introduce error or can deviate from standard protocols. Most assays require additional reagents or consumables prior to being read by an instrument. These supplies should be relatively inexpensive, and contribute to high sensitivity, accuracy, and reproducibility of the assay.

**Table 1:** Preferred characteristics of new diagnostic assays

Optimal criteria	Importance	Comment
Platform characteristics		
Low capital cost	Medium	Cost should fit clinical laboratory budgets
Ease of maintenance	Medium	User serviceable or low service contract cost
Ease of operation	High	No special skills outside of basic lab skills
Small footprint/portable	Medium	Not hardwired, ventilated, or plumbed
Expandable	Medium	Handles single or multiple samples, high throughput adaptable, assay reagents easily adjusted for sample number
Updateable	High	New organism profiles can be easily added to data libraries or assays by user or vendor
Versatile	Medium	Adaptable for other uses
High precision and accuracy	High	Assays should be reproducible with little variation and an accurate reflection of the true value (matches reference standard)
Data output	High	Fungus name or +/– result, no downstream analysis or data manipulation required
Input sample characteristics		
Broad input source (pure culture, human tissue or fluids, non-viable cells)	Low	Does not require organisms to be purified away from tissue, low interference from background material
Sample preparation part of platform	High	Standardized sample preparation with few or no user manipulations
Works on live or dead cells	Medium	Advantageous for fixed tissue samples
Works with yeast and mold morphologies	Medium	Assay or platform should not be affected by physical characteristics of culture
Assay characteristics		
Cost	High	Inexpensive reagents with a long shelf life, easy storage
Rapid turnaround time	Medium	Easily completed in single work shift, 3<h optimal
High sensitivity and specificity	High	Approach 100%
Positive and negative predictive values	High	Approach 1.0
Quantitative	Low	Allows clinicians to monitor treatment effectiveness
Detects and discriminates multiple analytes	Low	Able to output multiple identifications
Wide reportable range	Medium	Detects minute amounts but maintains linear response over broad range of target amounts
High analytical sensitivity	High	Demonstrates very low limit of detection

For any diagnostic assay to be successful in the clinical laboratory, ease of use and turnaround time are crucial. Assays that can be completed rapidly with minimal sample preparation are desirable. For fungal infections in particular, the ability to output quantitative values that help determine fungal load can provide clinicians with useful information for monitoring the effectiveness of treatment. Similarly, particularly for infected tissue, an assay should have a wide analytical range that allows it to be accurate with either minute or large numbers of cells. Unfortunately, as for all microorganisms, antifungal resistance is a growing problem. Thus, there is an increasing need for assays that can yield an identification and simultaneously determine if drug resistance is present.

Different platforms will have different specimen types that can be used. Whole-cell assays may be problematic due to the complex morphology of fungi (e.g., single-celled yeasts, filamentous hyphae, or both [dimorphism]). While yeast cells are usually handled by any instrument that uses whole-cell inputs, analysis of molds may be difficult or impossible due to physical problems posed by hyphae, which can clog channels or tubes, clump, or be unevenly distributed in liquid^36^. Platforms that work on dead or lysed cells are especially valuable due to the increasing need to perform diagnostic tests on fixed specimens. Unfortunately, formalin, typically used in the fixation process, can fragment and crosslink DNA and proteins^37^, inhibiting downstream steps of some assays, such as PCR. However, because of the importance of fixed samples, there have been recent improvements in extracting fungal DNA from formalin-fixed samples, such as by using an extended heating step^38,39^. Alternatively, coordinating with surgeons to obtain a section of tissue that is frozen but not fixed would alleviate much of this problem.

### Reference standards

The establishment of reference standards for assay validation is an obstacle that can be difficult to overcome. The fungi that a diagnostic test claims to identify must be validated with the assay^31,34^, so developers need to maintain or have access to a collection of fungi that covers the claimed targets. Type cultures, the original strain from which the fungal species description was generated, are the best references because they represent the standard of the species, are well-characterized, and are typically maintained in biocurated culture collections. However, obtaining them from culture collections may be difficult due to cost, proprietary concerns, or availability. Alternative well-characterized strains can suffice, however, there should be multiple isolates for each species. A collection of clinical isolates also should be included as strain variation is not unusual. For assays that are open-ended and can continually add new species for identification, validation using the reference strains and other well-characterized isolates must be performed. This necessity is particularly important for assays that integrate with linked databases such as DNA sequence-based and matrix assisted laser desorption/ionization-time of flight mass spectrometry assays (MALDI-TOF MS). Complicating this issue is the frequently changing fungal taxonomy and nomenclature. Fungi can have two completely different names if both a sexual phase (teleomorph) and an asexual phase (anamorph) have been identified. This dual naming system, which created confusion, has recently been changed to a one-fungus-one-name system^40^. However, it is imperative that clinicians and microbiologists keep up to date with any changes.

Unfortunately, fungal taxonomy and nomenclature still remain complicated issues as the application of molecular-based tools for species classification has caused extensive upheaval in fungal taxonomy due to the rapid discovery of new organisms, reclassification of known organisms due to new phylogenetic analyses, and perhaps the greatest problem, assigning new names to known fungi based on DNA sequence variations. From a clinical perspective, in some cases this specificity is important, such as when there is a difference in antifungal susceptibility as in the case on *Aspergillus lentulus*, a sibling species of *A. fumigatus* that displays slight differences in conidial structures but significant differences in susceptibility to clinically available antifungals^41^. Conversely, creating new species based solely on sequence variations without phenotypic differences is evolutionarily informative but clinically complex^42^. In fact, this problem has divided mycologists into “lumpers”, who are inclined to keep fungi with similar characteristics under the same name, and “splitters”, who are inclined to separate fungi based on slight sequence differences that may or may not be of clinical consequence^43^. Lumping multiple species or subspecies together into species complexes may be clinically preferred if every group member responds to the same antifungal at the same dose and is phenotypically similar. On the other hand, splitting fungi into more detailed taxonomic classifications provides useful evolutionary information, but also could provide useful clinical information by linking virulence factors or pathogenicity to a specific taxonomic classification. For example, *Exophiala dermatitidis* and *E. spinifera* are melanized, dermatiaceous fungi that display similar phenotypes. The general description of the type of disease they cause is phaeohyphomycosis, which includes many species of *Exophiala*. However, *E. dermatitidis* is a pulmonary colonizer that is often neurotropic while *E. spinifera* is not associated with these types of infections^44^. While there are important arguments in support of both strategies, for diagnostic assay development, keeping reference standards up to date is crucial for the accurate function of the assay.

### The approval process and clinical implementation

**Fig. 1:**
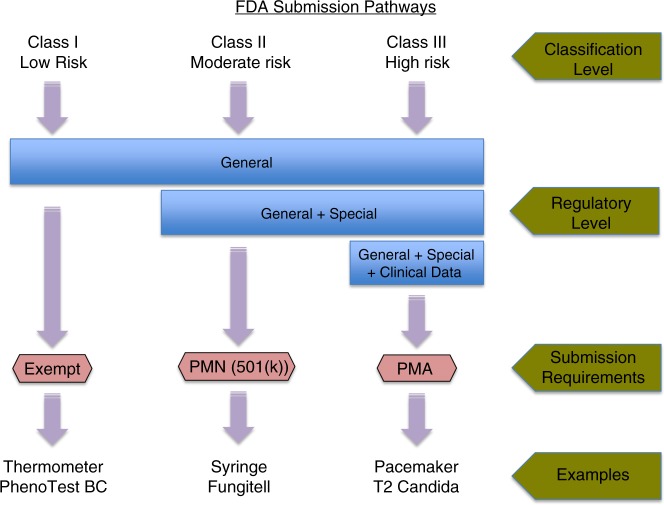
FDA approval pathways for medical devices. The approval process is based on risk to patients, with higher risk requiring more regulatory control. The FDA assists in the classification process through their database, which contains almost 2000 generic medical devices organized into 16 different panels. Classification is determined in part by location of the device on the patient, duration of device contact with the patient, and whether the device will be active or passive (active devices require power, usually electrical). Class I devices are the lowest risk and most are exempt from premarket notification. The device and company must be registered with the FDA but approval is not required. Class II devices need FDA clearance through the 510(k) premarket notification (PMN) process. Class III devices need premarket approval (PMA) and usually clinical trials. Examples of fungal devices (bold) are the PhenoTest BC (Accelerate Diagnostics, Inc.), which identifies *Candida albicans* and *Candida glabrata*, the Fungitell assay for fungal β-d-glucan (Associates of Cape Cod, Inc.), and the T2Candida assay (T2 Biosystems, Inc.), which identifies five species of *Candida* from blood cultures

Before an assay is approved or cleared for in vitro diagnostic (clinical) use, it must undergo regulatory scrutiny. In the United States, regulation is the responsibility of the FDA. In Europe, devices marked as Conformitè Europèenne In Vitro Diagnostic (CE-IVD) are cleared to be marketed for clinical diagnostic use^45^. The FDA classifies medical devices, including diagnostic assays, based on the level of control necessary to provide assurance of safety and effectiveness, with Class I having the least risk, Class II having moderate risk, and Class III posing the greatest risk. Class III devices require premarket approval (PMA) by the FDA, which is accomplished by demonstrating safety and effectiveness in clinical trials (Fig. 1). Unfortunately, PMA can be a lengthy and costly process. Class I and Class II devices do not require PMA but do require premarket notification (i.e., 510(k) submission), where they demonstrate that the devices are similar to one or more legally marketed devices unless they are exempt (which most Class I devices are). An example of a Class II device change is a modification to an FDA-cleared MALDI-TOF MS clinical database used in the identification of different microbes where new species are being included.

Laboratory developed tests (LDTs) are diagnostic assays that are developed, validated, and used primarily for in-house testing within individual laboratories^45,46^. In the field of infectious disease, LDTs, including molecular-based assays, are widely used due to the unavailability of FDA-cleared or CE-IVD marked assays. Thus, these assays help meet important clinical needs for which commercial assays are unavailable. LDTs are not currently required to go through the 510(k) clearance or PMA processes; however, laboratories that use LDTs must still validate them for accuracy, precision, and clinical utility. Unfortunately, this process may be difficult for rare pathogens due to the scarcity of material needed for this purpose. Additionally, there may be marked variability between laboratories in quality-assurance programs used to monitor their performance^45^. For both commercially available, cleared diagnostic assays and LDTs, the test results must be put into the context of the clinical picture for proper interpretation and patient care. As with all molecular-based assays in infectious diseases, the result must be correlated with the clinical picture or risk factors that the patient has for particular infections or drug resistance. Positive results in the absence of infection or risk factors should be interpreted with caution. Incorporating molecular-based assays into routine clinical practice is often limited by the lack of understanding of their true clinical sensitivity. This problem may be due to the limited sensitivity of cultures, which are often considered the standard for comparisons with molecular-based assays. Thus, it is unknown if a positive result from a molecular assay truly represents infection, colonization, or contamination if culture results are negative. Furthermore, clinical sensitivity molecular-based assays are influenced by the patient population under study, the biological specimens that are tested (e.g., whole blood, serum, sputum, BAL), as well as the particular fungal species/infection that is targeted.

## The bottleneck of sample preparation

Sample preparation is a significant issue for fungal diagnostics. While viruses, bacteria, and even some parasites circulate in high numbers in body fluid, which simplifies sample preparation due to high target abundance, fungi often are not found in blood or body fluids at sufficient levels for simple sample preparation. In fact, a positive blood culture is not considered a factor for the diagnosis of invasive aspergillosis because blood rarely harbors culturable elements^47^. Fungi also are problematic diagnostic targets due to their cellular organization, which includes a rigid cell wall that may be resistant to lysis, regardless of yeast or mold morphology. Any assay that requires access to nucleic acids, proteins, or other molecules not secreted or on the outside of the cell usually must address this issue with extraction steps that disrupt the cell wall, either by physical, chemical, and/or enzymatic methods. Consequently, a new diagnostic platform may require a companion extraction component or rely on another vendor’s extraction device.

The specimen source also plays a very important role in extraction strategy. If template is being prepared from pure cultures, the amount of DNA is not a limiting factor. Unfortunately, these templates require a time-consuming outgrowth step. For this reason, extraction methods that work directly on clinical specimens are in high demand, but can be challenging for multiple reasons. Even with efficient extraction protocols, low or uneven amounts of fungal elements are often found in sample specimens and clinical specimens can swamp fungal targets with host nucleic acids or proteins. Body fluids that are easy to access, such as blood and urine, often do not contain any fungal elements at all, or fungi may only be present during certain clinical conditions (i.e., fungemia) in spite of an active infection. Interestingly, there is growing evidence that cell-free fungal nucleic acids are present in serum at levels suitable for nucleic acid detection, although it is not clear how they are protected from degradation^48^. Therefore, whole blood many not be the best substrate to assay for the presence of fungi^49^. Instead, different blood components may be better sources for fungal detection. In some cases detection of *C. albicans* is positive more often from serum than from blood^50^. However, consensus regarding the best blood component to assay for fungal diagnostics has not been reached. Other fluids, such as sputum, are not sterile and may contain other fungal species, many of which are inhaled but cannot grow at body temperature, which illustrates the problem of distinguishing a colonizer from an invader. Therefore, detection of fungi alone may not be enough to diagnose infection. Some estimate of fungal burden may be needed in order to distinguish colonization from true infection. Alternatively, a confirmed diagnosis may require multiple positive test results on samples collected at different times. An assay that provides fungal burden as an output could also be used to follow the course of infection and the effectiveness of treatment.

## Diagnostic platforms

Multiple technologies have been successfully used for fungal diagnostics, with many being developed into commercial assays. The diversity of technologies suggests that none have yet dominated the market. However, a single platform that serves all diagnostic needs may not be possible for various reasons. Several current methodologies are listed in Table 2, along with what we feel are their main strengths and weaknesses.

**Table 2:** Strengths and weaknesses of different platforms

Methodology	Strengths	Weaknesses
PCR	Amplifies low target amounts High sensitivity and specificity Can be quantitative Rapid (2–3 h) Multiplexable (multiple targets) Diverse platforms and methodologies Inexpensive instrumentation and assay Testing directly from specimens Sample-to-answer capability Small instrument footprint, portable	Requires purified template Moderate complexity a priori target selection required Contamination prevention is crucial Moderate technical expertise
Sequence	Highest specificity Inexpensive assay Panfungal capability Result in ~8 h Can detect new species Massive public databases Numerous commercial service vendors	High capital costs (for in house instrument) High complexity Requires PCR amplification step Template quality crucial to assay Data interpretation may be needed High technical expertise Singleplex assay, but multi sample
Whole-genome sequence	Highest specificity Identity, epidemiology, drug resistance Direct specimen testing Multi-or megaplex capable Tolerates mixed or contaminated samples Panfungal capability	High capital cost Additional supportive equipment Highest complexity Highest technical expertise Substantial analytical burden Extensive sample preparation Expensive per assay cost Massive data requires infrastructure Slow turnaround (days)
Hybridization	Multiplex to megaplex capability Diverse platforms Mixed analyte capability (ID and drug resistance genes, multiple microbe ID) Numerous systems FDA approved Result is +/– Result <8 h	Can be high complexity Can be multistep (PCR+ hybridization+detection) Variable capital cost Moderate technical expertise
Mass Spectroscopy	Results in minutes Easy to moderate sample preparation Result is +/– Capability could be panfungal	High capital costs High complexity Requires pure culture Singleplex only Limited databases (currently)

### Polymerase chain reaction

Of all the different platforms, PCR is the most diverse, with many different strategies that use amplification of a nucleic acid target as a basic component. This core technology is one of the most basic and widespread techniques used in molecular biology. Consequently, PCR is generally easy to employ and usually relies on inexpensive instrumentation. PCR also is among the fastest assays to yield results, and outputs can be quantitative and straightforward to interpret. These attributes have led to automation and adaptation to high throughput platforms. A major drawback of PCR as a fungal diagnostic platform is that there needs to be some level of suspicion that the fungus targeted by the assay is in the patient specimen as most PCR applications do not have a panfungal discriminatory capability. The specific nature of a PCR reaction enables it to be used directly on clinical specimens containing large amounts of host nucleic acid without obtaining pure fungal cultures in vitro. Unfortunately, a universal limitation of PCR for fungal diagnosis is the generally low amount of fungal DNA in clinical samples compared to viral or bacterial loads^48^. This limitation has driven, in part, the search for variations to enhance PCR sensitivity.

One of the earliest PCR variations was the ability to see results in real-time (real-time PCR or quantitative real-time PCR; qPCR). qPCR instruments display the output graphically in real-time as amplification cycles proceed. Output is typically detected by fluorescence, which can be as simple as measuring the amount of double stranded product, or adding a probe to the reaction that is specific for a target sequence within the amplicon. Multiple probes can be employed to create multiplex reactions. The wide use of qPCR has produced competition in all areas of the assay, including polymerases, probe chemistries, and instrument platforms. Virtually all of the major human fungal pathogens have multiple publications describing species or even strain-specific primers and probes, so it is possible to order an individual assay containing these specific reagents as a single kit.

While the most common forms of PCR employ thermocycling, which cycles through a broad range of temperatures (from 5 to 95 °C), a simplified form of PCR, termed isothermal PCR, does not require thermocycling and therefore does not rely on complex instrumentation nor thermostable polymerases. Isothermal reactions utilize a single temperature throughout the amplification process. Strategies that use this approach are particularly attractive to developing countries because instrument cost, complexity, and portability are greatly alleviated by using an incubator instead of thermocycler. Other PCR components are relatively inexpensive, which together, allow PCR to be used in regions of low income and poor infrastructure.

Loop Mediated Isothermal Amplification (LAMP), an isothermal PCR technique, is carried out at 60–65 °C and utilizes a polymerase that displaces one of the DNA strands as a new strand is synthesized. LAMP also uses four primers instead of the usual two, which provides a greater specificity than the typical pair of primers, although primer design is more challenging. LAMP has been applied to many of the major fungal pathogens^51–53^. A second type of isothermal amplification method used for fungal identification is called Nucleic Acid Sequence-Based Amplification (NASBA), which uses RNA as the template. This reaction typically is done at 41 °C and is extremely sensitive because the RNA target can be present in thousands of copies. In a recent study of invasive aspergillosis for example, Zhao et al. found NASBA to correlate well with an FDA-approved serum 1,3-β-D-glucan kit^54^. A third type of isothermal amplification method used for fungal identification is Rolling Circle Amplification (RCA). RCA uses a viral polymerase to amplify the target sequence in the form of a circular template. The major advantages of this method are that it can amplify targets to an excess of 10^9^ copies, requires little optimization, and is resistant to contamination, which is a major concern for other PCR reactions^55^. RCA has been successfully used with the Mucorales^56^, *Cryptococcus*^57^, and *Exophiala* species^58^ to name a few.

### DNA sequencing

**Fig. 2:**
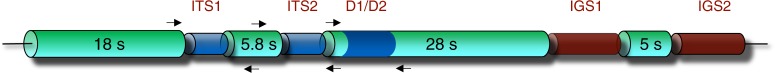
DNA sequence encoding fungal ribosomal RNAs. The genes for fungal rRNAs are organized as a repeating unit. A single unit consists of the sequence encoding the 18S rRNA (for the small ribosomal subunit), internal transcribed spacer 1, sequence encoding the 5.8S rRNA, internal transcribed spacer 2, sequence encoding the 28S rRNA (for the large ribosomal subunit), intergenic sequence 1, sequence encoding the 5S rRNA, and intergenic sequence 2. The 18S, ITS1, 5.8S, ITS2, and 28S sequences are transcribed as a single RNA, which is then spliced to remove the ITS1 and ITS2 regions (blue cylinders) prior to assembly with the 5S rRNA into the complete ribosome consisting of the 18S, 5.8S, 28S, 5S (green cylinders) and assorted proteins. The ITS1 and ITS2 regions make up the ITS sequence, which is generally ~600bp in length although it can vary by more than 200bp. The D1/D2 region (blue shaded region on 28S) is similar in length. Both regions are informative with regard to sequence identification although the D1/D2 region is usually more conserved. Priming sites for PCR are shown as black arrowheads and can be used in various combinations. The 5S sequence is transcribed separately and is flanked by the two IGS regions (brown cylinders), which can be very informative, however, they vary tremendously in size, making amplification by PCR difficult

Sequence-based identification of fungi is arguably the “gold standard” of fungal molecular identification^59^. This strategy is an extremely useful diagnostic tool due to the wealth of information found in public databases, such as GenBank. In addition to individual genes and genomes, GenBank contains an extensive number of ribosomal RNA gene (or rDNA) sequences, with more than 500,000 records for ITS1 and 28S deposits. Using the fungal rDNA as a diagnostic target was made possible mainly through the work of White et al., who used universal priming sites on DNA sequences encoding rRNAs of the small and large ribosomal subunits (18S and 28S rRNAs) to amplify across the ITS region^60^ (Fig. 2). Additionally, ribosomal RNA genes in eukaryotes are present in multiple copies, which confers added sensitivity during the PCR reaction. Finally, GenBank is both broad and deep with regard to fungal rDNA sequence information, from almost 5000 species. The most common fungi may have more than one hundred ITS sequence deposits and even rare fungi are often accompanied by an ITS sequence deposit, which is vitally important for the identification of new fungal pathogens. In addition to the ITS sequence, there is a variable region within the 28S rDNA that also confers some diagnostic value. This region, called the D1/D2 region^61^, is not as sensitive as the ITS region and in many cases can only provide genus-level discrimination; however, it served as one of the earliest sequencing targets for a commercial diagnostic assay (MicroSEQ D2 rDNA Fungal Sequencing Kit, Thermo Fisher, Grand Island, NY).

While DNA sequencing is broadly used by many investigators and many reference laboratories, it is not widely commercialized due to the large capital investment and expertise needed to run a sequence-based assay system. In many cases, the ITS region is not sensitive enough to discriminate between some fungi at the species level, which necessitates using other loci to reach species level identification. This option may be clinically relevant for certain species complexes where differences in antifungal susceptibility and clinical outcomes have been reported between sibling species (e.g., *Aspergillus fumigatus* species complex and *Scedosporium apiospermum* species complex). Thus, identification to the species level is clinically relevant in certain instances but not necessarily in others (e.g., *Candida parapsilosis* species complex)^41,62–64^. However, this additional step may result in diagnostic delays. It is also not possible to rely solely on GenBank as a clinical database due to its open nature. For this reason, GenBank is error prone and requires expertise both in bioinformatics and mycology in order to accurately extract good information from fungal search outputs^65^. However, for fungi that are not extremely rare, it is possible to search GenBank using customized filters to enhance hit output reliability. For example, reference sequence databases (RefSeq) contain highly annotated deposits that can be exclusively searched, or searches can be conducted for type material deposits using the limit function checkbox. More specific searches, such as searches only against culture collection deposits, can be done using Entrez query syntax in the Entrez query-specific search set. An alternative to the NCBI site are smaller biocurated databases that allow BLAST searches, but which contain verified sequences (see Prakash et al. for review^66^). Consequently, a commercialized system may require a closed, biocurated database that can be established using reference sequences from GenBank or other databases, or by generating their own sequences.

### Whole-genome sequencing

A rapidly developing alternative to rDNA sequencing is whole genome sequencing (WGS). However, even with falling costs, this technology is still too expensive, complicated, and too slow for routine use in most clinical microbiology laboratories. In fact, the complexity and amount of data produced by WGS technology can actually be an impediment on the utility of the information^67^, at least until bioinformatic platforms catch up to the sequencing platforms in throughput. The value of WGS lies beyond simple identifications. WGS can provide epidemiological information about each strain, and the potential for drug resistance, all in the same run and without *a priori* suspicion of what fungal species might be present^68,69^. Surveillance and epidemiological applications would have the potential to precisely pinpoint the source of a specific strain as well as track its spread. Each of these capabilities can be determined across multiple target species that could range from viral, to bacterial and fungal^70^, making runs more economical as they can be performed at regular intervals with numerous samples. However, an important factor in cost can be genome size, which is enormously variable in fungi (8.97–177.57Mb)^71^, or genome dynamics. A low degree of nucleotide diversity, such as in some species of *Penicillium*, can also limit epidemiological use^72^. WGS has tremendous value in metagenomic analysis. Non-sterile body sites, in contrast to pure cultures that may be used for screening of phenotypic markers or epidemiology, are complex ecosystems of microbes that can be sampled for the presence of fungi. WGS has already been applied to metagenomic analysis of the human mycobiome in a variety of ways. Ghannoum et al. have used WGS to characterize the human fungal oral mycobiome^73^, and WGS has been used to identify a specific species of fungus in an intriguing link between fungi and Crohn’s disease^74^.

WGS is now moving from the research arena to clinical laboratories so its capabilities as a frontline infectious disease diagnostic tool are not yet fully recognized. With advances in instrumentation, bioinformatics, and protocols, WGS will almost certainly find further uses in clinical microbiology laboratories. In fact, fourth-generation sequencing technology has now arrived and uses nanopore technology to generate sequences based on detecting changes in electrical conductivity. These changes occur as DNA molecules are threaded through a biological pore that is embedded in a biological membrane or formed in a solid-state film^75^. While the basic technology has been known for more than 20 years, early platforms were recently developed by Pacific Biosciences Inc. and Helicos Biosciences. Subsequently, Oxford Nanopore Technologies introduced their version of this technology in 2014 (MinION sequencer)^76^. The major weakness with nanopore sequencing has been its error rate, which can range from 12% to 35%^77^, and makes this technology less accurate than current WGS platforms. However, the technology has yielded some important advances in instrumentation. The MinION instrument is portable and roughly the size of a computer memory stick. It can interface with computers easily through a USB port making data transfer simple. Read lengths using the MinION device have been recently reported to >800kb^78^. Perhaps the most exciting aspect of this technology is the lack of complicated library preparation, which is a current bottleneck of WGS. Template DNA for nanopore sequencing can be prepared in under an hour and human genome-length sequencing runs can theoretically be completed in 15 min^79^. Reducing the error rate remains an intense research focus as compensation for accuracy issues requires extensive downstream analysis using specific algorithms. Nonetheless, the portability of these instruments, ease of sample preparation, and sequencing speed potentially could make WGS technology a point of care molecular diagnostic strategy.

### Mass spectrometry

One of the fastest growing diagnostic platforms that is already being integrated into clinical laboratories is matrix assisted laser desorption ionization-time of flight mass spectrometry (MALDI-TOF MS) (reviewed in ref. ^80^). MALDI-TOF MS uses a laser to ionize biomolecules, which are then detected and measured based on their mass-to-charge ratio. The time-of-flight (TOF) component generates a peptide mass fingerprint that is used to interrogate a database. MALDI-TOF MS has a number of strengths that fit within a clinical laboratory environment. It is fast, providing an identification in minutes, and also requires minimal sample preparation. The database that is searched with the unknown fingerprint is typically closed but can accept new identifications by instrument users if the assay will not be used under FDA-approved conditions. User expanded databases must follow stringent validation requirements. The major weakness of MALDI-TOF MS is the need to generate analytes from pure cultures, which can add days to the turnaround time, although testing on direct clinical specimens is a major research focus for expanding platform capabilities^81^. There also can be some fingerprint variation depending on culture conditions, which can be difficult to standardize for fungi^82^. However, MALDI-TOF MS has had good success identifying several major human fungal pathogens, including *Aspergillus, Candida, Cryptococcus*, and *Fusarium* species, the Mucorales, dimorphic fungi (*Histoplasma capsulatum*, *Blastomyces dermatitidis*, and *Coccidioides* species), and some dermatophytes to name a few^80,83,84^.

Electrospray ionization-mass spectrometry (ESI-MS), like MALDI-TOF MS, is a mass spectrometry-based method that can be used for fungal characterization or identification. While different biomolecules can be analyzed using this instrumentation, fungal diagnosis is typically performed by analyzing nucleic acids. Because this platform can rapidly (~6 h) identify a large number of different fungi and is database driven, *a priori* suspicion is not needed. However, amplicon contamination was found to be possible without suitable structural modification and per assay costs were somewhat expensive^85^. Early versions of the fungal database also yielded misidentifications^85^. Alternatively, direct testing on clinical specimens may be possible^85^, and this capability may convey an advantage for this platform over that of MALDI-TOF MS. Furthermore, ESI-MS can accept multiple specimens in a single run, and the PCR component enhances sensitivity for low abundance organisms^86^. However, it is unclear whether or not this technology will have long term viability in clinical laboratories. A commercial version (The IRIDICA system:formerly PLEX-ID, Abbott Inc., Abbott Park, Il.) that had good clinical performance and was CE-IVD marked for use in Europe was recently discontinued^87^.

### Hybridization

Numerous assays based on hybridization have been developed for fungi with several already commercialized. Fluorescence in situ Hybridization (FISH) is an oligonucleotide probe-based, cytogenetic assay that targets rDNA sequences^88^. The key aspect of this assay is the diffusion of the fluorescently labeled probe across the cell wall and membrane, with fluorescent output detected microscopically after the probe anneals to its target. Specimens need little preparation, which circumvents the sample preparation bottleneck and contributes to the quick assay turnaround time. Variations of the assay include Peptide Nucleic Acid (PNA) FISH, which utilizes a peptide nucleic acid as the probe, leading to stronger binding and more efficient reactions due to the uncharged, neutral backbone^89^. Because this assay is fast, can be performed directly on clinical specimens such as blood, and requires little sample preparation, it has been in use for a number of years for the detection of *Candida*. AdvanDX (Woburn, MA) has two FDA-cleared PNA-FISH products on the market, Yeast Traffic Light FISH and *Quick*FISH. PNA-FISH has also been used to identify several other fungal species, including *Aspergillus*, *Fusarium*, and *Scedosporium* species^90^.

Multiple array-based hybridization strategies have been developed over many years and typically work by using a PCR amplicon as a hybridization probe for an array of targets. One of the advantages of these methods is that targets can be as simple as individual species-specific oligonucleotides, which is inexpensive but also allows a large number of species to be identified at once. The simplest variations of this process bind oligos to a solid matrix such as glass or a membrane, which is then used for the hybridization. The Luminex xTAG Fungal Assay (Luminex Molecular Diagnostics, Toronto, Canada) is a more complex variation of this theme. This assay can detect up to 23 different species of fungi based on the xTAG technology^91^. Identification of different species occurs after a PCR step to amplify unknown fungal nucleic acid. Species-specific beads are added after the PCR reaction and bind to short specific primer sequences on the amplicon. The sample is then read with a laser to identify individual bound tags, which results in an identification.

### Magnetic resonance

Magnetic resonance is not widely used in molecular diagnostics. However, one assay has been developed and FDA-approved for diagnosis of common *Candida* species (T2Candida®). This platform (T2MR, T2 Biosystems, Inc., Lexington MA) combines PCR and magnetic resonance by mixing magnetic nanoparticles with an ITS2 amplicon. Magnetic bead-bound amplicons change the local H_2_O environment, which alters the magnetic resonance signal. The signal can then be detected and measured to yield a species-specific output. This assay displays a number of preferred molecular diagnostic assay characteristics, including analytical sensitivity >91%, specificity >99%^92^, and rapid turnaround time (3–5 h). The T2Candida system is fully automated and closed, with an extraction module included as part of the platform. However, only 5 common *Candida* species are targeted (*C. albicans, C. glabrata*, *C. parapsilosis*, *C. tropicalis*, and *C. krusei*) and the amplicon consists of only the ITS2 region, which may sacrifice some specificity. The assay is unable to distinguish between *C. albicans* and *C. tropicalis* nor between *C. glabrata* and *C. krusei*. Instead, *C. albicans* and *C. tropicalis* are grouped together for identification due to the similarity in antifungal susceptibility profiles for these species, as are *C. glabrata* and *C. krusei*^93^. The PCR amplification step necessitates an index of suspicion before deploying the test; however, the ITS2 region falls between two conserved primer binging sites (5.8S rDNA and 28S rDNA), which can provide flexibility for species detection beyond *Candida*. Because of the location and nature of the PCR amplification, it is possible that the assay could be expanded to other fungi.

## Commercial assays

Numerous molecular-based diagnostic assays are commercially available and are either FDA-cleared or CE-IVD marked for clinical use in the United States and Europe, respectively (Table 3). Several different technologies are used in these assays, including chemiluminescent labels, single-stranded DNA probes, PNA-FISH, real-time PCR or nested multiplex PCR (nmPCR) with melt-curve analysis, and DNA amplification followed by magnetic resonance. In addition, two commercially available MALDI-TOF MS systems are also cleared for fungal species identification in clinical diagnostic testing. The commercially available and cleared assays primarily focus on the detection and identification of common *Candida* species, including *C. albicans, C. glabrata, C. krusei, C. parapsilosis*, and *C. tropicalis*, but some are also able to detect and identify *Cryptococcus* or *Aspergillus* species. The chemiluminescent AccuProbe assays detect dimorphic fungi (i.e., *Coccidioides*, *Blastomyces*, and *Histoplasma*). Most commercial assays are designed to detect and identify the fungi associated with infection. However, one assay, the AsperGenius assay is also able to identify prevalent mutations in the *CYP51A* gene that are associated with resistance to the triazole class of antifungals^94^. Some studies have also reported the use of MALDI-TOF MS for antifungal susceptibility testing, although promising results have not been consistently reported^95–97^.

**Table 3:** Examples of molecular assays for fungal infections with FDA clearance for clinical use or CE-IVD marked for use in Europe

Assay (manufacturer)	Assay methods	Advantages	Limitations
AccuProbe Coccidioides, Blastomyces, and Histoplasma Culture Identification Tests (Hologic)	Chemiluminescent labeled, single-stranded DNA probe	•Rapid identification of dimorphic fungi, including *Coccidioides, Blastomyces*, and *Histoplasma* •Sensitivity/specificity—*C. immitis/C. posadasii* 98.8%/100%; *H. capsulatum* 100%/100%; *B. dermatitidis* 98.1/99.7%	•Requires growing culture of fungus •False positives with other fungi
Yeast Traffic Light and Quick FISH (AdvanDx)	PNA-FISH	•Relatively fast turnaround time from positive blood culture bottle •Sensitivity/specificity— 92.3%–100%, 94.8–100%^98^	•Requires positive blood culture bottle •Limited number of *Candida* species detected •Requires fluorescent microscope
BioFire Film Array (bioMerieux)	Nested multiplex PCR with DNA melt-curve analysis	•Blood culture ID (BCID; for *Candida* species) and meningitis/encephalitis (ME; for *Cryptococcus*) panels available •ME panel can detect *Cryptococcus* directly in CSF •Sensitivity/specificity for BCID —96.7%–100%, 99.8%–100%	•Requires positive blood culture bottle for BCID •Limited number of *Candida* species detected in BCID assay •High equipment and reagent costs
T2Candida (T2Biosystems)	PCR with nuclear magnetic resonance	•Can be performed directly on blood •Fast turnaround time from collection of blood •High analytical sensitivity •Sensitivity/specificity—91.1%, 99.4%	•Limited number of *Candida* species detected •High equipment and reagent costs
SeptiFast LightCycler (Roche)	Real-time PCR with DNA melt-curve analysis	•High analytical sensitivity •Detects five common *Candida* species and *Aspergillus* •Sensitivity and specificity are calculated based on combined results for bacterial and fungal cultures	•Limited number of *Candida* species detected •Not currently available for clinical use in US
AsperGenius (PathoNostics)	Multiplex real-time PCR	•Detects *Aspergillus* directly in BAL specimens •Can also detect mutations in *CYP51A* gene associated with azole resistance •Sensitivity/specificity—88%, 77.8%^99^	•Limited number of *Aspergillus* species detected •Not currently available for clinical use in US
MycAssay *Aspergillus* (Myconostica)	Real-time PCR with molecular beacons	•Eighteen different *Aspergillus* species •Detects *Aspergillus* directly in serum and BAL specimens •Sensitivity/specificity—70%, 90.5%^100^	•Not currently available for clinical use in US

Clinical sensitivity and specificity were obtained from assay information provided by vendor or by publication as indicated.

Several of these assays require positive blood culture bottles due to the lack of analytical sensitivity when performed on direct specimens. These include the Yeast Traffic Light and QuickFISH PNA-FISH assays, and the BioFire Film Array Blood Culture ID assay for *Candida*. Others can be performed on direct specimens, such as CSF (BioFire Film Array for *Cryptococcus*), blood (T2Candida), serum (MycAssay *Aspergillus*), or BAL fluid (MyCAssay *Aspergillus* and AsperGenius assay). The two MALDI-TOF MS systems as well as the AccuProbe assays for dimorphic fungi currently require a pure culture of the infecting pathogen.

## Conclusions

It is difficult to predict which of the molecular platform technologies will become the major molecular diagnostic strategy for fungi. While a one-size-fits-all assay probably represents the holy grail of diagnostic microbiology, for fungi, current research will likely focus on multiple approaches, one to detect common fungal pathogens, including *Candida* and *Aspergillus* species, and one to detect less common causes of invasive mycoses. Embedded within these approaches is the desire for sample-to-answer platforms that remove as much technician input as possible. The unique problem that many fungi present with regard to pre-assay processing will always make sample preparation a much larger issue for fungal molecular diagnostics than it is for virtually all other microbes. Unfortunately, there may be some infections in which targeting the fungus will never be sensitive enough because fungal elements cannot be recovered from specimens. These situations insure that alternate assays, such as serology-based assays, are always present in the clinical laboratory diagnostic assay portfolio. Unfortunately, cost is always a driving factor for clinical diagnostics, both with regard to instrumentation as well as individual per assay costs, not to mention labor that may be associated with running the assay. While cost concerns can be somewhat mitigated if fungal identification is done at a reference laboratory, shipping specimens to another laboratory delays the time until results are available to clinicians, which may adversely affect patient outcomes. Consequently, new assays will need to take into account these factors regardless of how sensitive and specific they may be. In spite of these challenges, molecular diagnosis of fungal infections is becoming an important and increasingly common diagnostic practice that works in conjunction with, and in some cases may replace, live culture as the main diagnostic strategy in the clinical laboratory.
